# Reviews

**Published:** 1861-11

**Authors:** 


### REVIEWS.

*The Morbid Effects of the Retention in the Blood of the Elements of the Urinary Secretions. Fiske Fund Prize Essay. By* William Wallace Morland, M. D.*: Philadelphia,* Blanchard & Lea.

"No organs in the human body play a more important part in the economy of life and health than the kidneys—their office is *the depuration of the blood.* In however slight a degree their function is interfered with some untoward effects are produced. These may often be barely noticed and easily recovered from ; in many instances, however, although disregarded at first, they are sure of their ground, hard to be dislodged, and too frequently insidious and widely and surely destructive. The more open and overwhelming attacks of disease, which, by rapidly disabling the kidneys and extensively injuring their tissue, at once and distinctly tell upon the constitution, reveal in plain characters the close connection between the vital torrent and its purifying agents.

The subject, as proposed by the Trustees of the Fiske Fund, necessitates, *first,* the enumeration of "the elements of the urinary secretion ;" and *secondly,* the recital of the effects produced by the undue "retention" of each of them in the blood.

By the expression "elements of the urinary secretion," as here used, we understand its constituents in a state of health. These constituents, by a vital law, are to be eliminated from the blood ; and their retention therein, beyond a certain time, will certainly cause "morbid effects."

The following enumeration of the urinary elements is taken from one of the latest and most reliable authorities. The analysis is made up from an average of the composition of all the urine passed in twenty-four hours. *Average quantity* from twenty-four hours, 1400 to 1600 cubic centimetres ; 49 to 56 fluid ounces. *Average specific gravity*, 1.020. *Mean amount of solids*, 55 to 56 grammes (a gramme is 15.4440 grains, English.)

*Constituents.*

| | | | |
|---|---|---|---|
| Water . . . . . . | 1345 to 1534 grammes. | | |
| Urea . . . . . . | 39 to 40 | " | 463 to 617 grs. |
| Uric Acid . . . . . | 0.5 | " | or 7.5 " |
| Creatine . . . . . | 0.3 | " | or 4.5 " |
| Creatinine . . . . . | 0.45 | " | or 0.5 " |
| Sarkine, Unræmatine, Uroxanthine } . . . | undetermined. | | |
| Hippuric Acid . . . . | 0.5 | " | or 7.5 " |
| Chlorine . . . . . | 6 to 8 | " | 92 to 123 " |
| (or Chloride of Sodium . . | 10 to 13 | " | 154 to 200 ") |
| Sulphuric Acid . . . . | 1.5 to 2.5 | " | 23 to 38 " |
| Phosphoric Acid . . . . | 3.66 | " | 56 " |
| Potash and Soda, Lime and Magnesia } . . | undetermined. | | |
| Earthly Phosphates . . . | 1.28 grammes, | | 19 " |
| Iron . . . . . | undetermined. | | |
| Ammonia . . . . . | 0.7 grammes, | | 10 " |
| Trimethylamine, Carbonic Acid, Phenylic Acid, Damaluric Acid } . . . | undetermined. | | |

"The minor estimates account for 48 out of 55 grammes of solids, the larger estimates for 62 out of 66 grammes of solids."—THUDICHUM, *op. cit.*

From an examination of the above table, in connection with the requisitions of the subject, it will be evident that we have only to indicate the pathological effects arising from the undue retention in the blood, of the following constituents of the urinary secretion : Water ; Urea ; Uric Acid ; Creatine ; Creatinine ; Hippuric Acid ; Chlorine ; Chloride of Sodium ; Sulphuric Acid ; Phosphoric Acid ; Earthy Phosphates, and Ammonia. The other ingredients of the urine, mentioned as being found in "undetermined" proportions, cannot enter into the list, in a practical consideration of the subject."

We have thus far quoted the author, for the purpose of showing our readers something of the general character of this Essay, and to give the topics which are discussed in a very full, comprehensive, and complete manner.

The author shows a most familiar acquaintance with the various subjects brought under consideration, has quoted largely and fully what has been written by others, and has expressed his own views in a clear, plain, scholarly style, making a valuable book of near 100 pages, which should be in the hands of every physician who desires to possess the gist of the whole matter in a readable book, of practical value.

